# Quality of Vegetable Oil Prior to Fortification Is an Important Criteria to Achieve a Health Impact

**DOI:** 10.3390/nu6115051

**Published:** 2014-11-11

**Authors:** Nuri Andarwulan, Desty Gitapratiwi, Arnaud Laillou, Dwi Fitriani, Purwiyatno Hariyadi, Regina Moench-Pfanner, Drajat Martianto

**Affiliations:** 1 Department of Food Science and Technology, Faculty of Agricultural Engineering and Technology, Bogor Agricultural University, P.O. Box 220, IPB Darmaga Campus, Bogor 16680, West Java, Indonesia; E-Mails: fi3riani.dwi@gmail.com (D.F.); hariyadi@seafast.org (P.H.); 2 Southeast Asian Food and Agricultural Science and Technology (SEAFAST) Center, Bogor Agricultural University, IPB Darmaga Campus, Bogor 16680, West Java, Indonesia; E-Mails: destygitapratiwi@gmail.com (D.G.); drajat_martianto@yahoo.com (D.M.); 3 United Nations Children’s Fund (UNICEF), No. 11 Street 75, Sangkat Sraschark, Phnom Penh 12201, Cambodia; E-Mail: alaillou@unicef.org; 4 Global Alliance for Improved Nutrition, 354 Tanglin Road #03-13/14, Singapore 247672, Singapore; E-Mail: rmoenchpfanner@gainhealth.org; 5 Department of Community Nutrition, Faculty of Human Ecology, Bogor Agricultural University, IPB Darmaga Campus, Bogor 16680, West Java, Indonesia.

**Keywords:** unbranded bulk palm oil, vitamin A fortification, thermal oxidation, PV, shelf life

## Abstract

Unbranded palm cooking oil has been fortified for several years and can be found in the market with different oxidation levels. This study aimed to investigate the stability and shelf life of unbranded, bulk, vitamin A-fortified palm oils with the most commonly observed oxidation levels in Indonesia. Three types of cooking oils were tested: (i) cooking oil with a peroxide value (PV) below 2 mEq O_2_/kg (PO1); (ii) cooking oil with a PV around 4 mEq O_2_/kg (PO2); and (iii) cooking oil with a PV around 9 mEq O_2_/kg (PO3). The oil shelf life was determined by using accelerated shelf life testing (ASLT), where the product was stored at 60, 75 and 90 °C, and then PV, free fatty acid and vitamin A concentration in the oil samples were measured. The results showed that PO1 had a shelf life of between 2–3 months, while PO2’s shelf life was a few weeks and PO3’s only a few days. Even given those varying shelf lives, the vitamin A loss in the oils was still acceptable, at around 10%. However, the short shelf life of highly oxidized cooking oil, such as PO3, might negatively impact health, due to the potential increase of free radicals of the lipid peroxidation in the oil. Based on the results, the Indonesian government should prohibit the sale of highly-oxidized cooking oil. In addition, government authorities should promote and endorse the fortification of only cooking oil with low peroxide levels to ensure that fortification is not associated with any health issues associated with high oxidation levels of the cooking oil.

## 1. Introduction

Indonesia is a leading global supplier of cooking oil [1]. Domestically, fortification of unbranded cooking oil has proven to be the most suitable food vehicle to increase vitamin A intake on a pilot and large-scale basis in Indonesia [2]. Fortification of cooking oil with retinyl palmitate, which reaches 94% of Indonesian families, will ensure that women will receive 54% and children 51% to 57% of their estimated average requirement (EAR) for vitamin A [2]. As highlighted by Soekirman *et al.* [1], most of the cooking oil industry in Indonesia has expressed a desire to start fortification of cooking oil, since the additional cost to the industry is limited (US$1.71/Metric Tonnes). However, even as cooking oil fortification with retinyl palmitate is recognized as cost-effective and simple to implement [3], the quality of cooking oil (assessed by peroxide values) prior to fortification has been highlighted in a recent study from Egypt [4] as a potential barrier to ensuring the stability of retinyl palmitate. The oxidation level greatly interacts with the stability of vitamin A added to the oil. Vitamin A oxidizes faster and loses its activity in the presence of highly-oxidized oils (with a high level of peroxide) [5].

The Egyptian study found an increase in vitamin A decay once the peroxide levels in oil were more than 2 mEq of active oxygen per kilogram and recommended that before any national fortification program is implemented, the losses of vitamin A due to the quality of cooking oil be estimated. A 2011–2012 SEAFAST (Southeast Asia Food and Agricultural Science and Technology Center) study on the quality of unbranded bulk palm cooking oil (to be referred to as “cooking oil” in this document) in Indonesia showed that the quality of cooking oil at the point of manufacture and at the distributor was inconsistent. For example, the peroxide value (PV) of cooking oil analyzed ranged between 0 and 8.94 mEq O_2_/kg [6].

Although a medium-scale project of fortified cooking oil is already on-going and has shown consumer acceptance in Indonesia [1], there have been no studies undertaken in Indonesia to evaluate the stability of added vitamin A in cooking oil, especially when stored at various temperatures. Therefore, the aim of this study was to investigate the shelf life of cooking oil and the stability of vitamin A added to cooking oils with three different qualities as defined by peroxide content. The findings have implications for (i) the amounts of vitamin A to be added by the producers in order to ensure that the cooking oil is fortified following the national guidelines and (ii) the possibility of fortifying highly-oxidized cooking oil.

## 2. Experimental Section

The shelf life of oil was determined by using accelerated shelf life testing (ASLT), where the product is stored at elevated stress conditions (storage temperature of 60, 75 and 90 °C). Oil samples were stored using an amber bottle, and after the removal of oxygen in the headspace volume by flushed nitrogen, the bottles were sealed and kept in a dark room (inside an incubator) to avoid direct exposure from light. Periodic oil sampling for analyses was conducted at several time points of measurement. Each sample for analyses was taken from a different amber bottle. The temperatures were selected to stimulate relatively fast degradation, to determine the shelf life using ASLT (without destroying the fundamental characteristics of oil) and the Arrhenius model. PV, free fatty acid (FFA) and vitamin A concentration were measured in an accelerated storage test [7]. All laboratory analyses were performed in triplicate, and averages are presented.

### 2.1. Analysis of the Oxidative Status of Cooking Oil

Fortified cooking oil samples were analyzed for the following parameters: (i) PV; and (ii) FFA level. These two parameters are the most common parameters to characterize oil deterioration [8]. To measure PV, the AOCS Cd 8–53 method [9] was used: 5 ± 0.5 g of oil was dissolved in 30 mL of glacial acetic acid (chloroform solution). After the addition of 0.5 mL of saturated potassium iodide with occasional shaking for 1 m and 30 mL of distilled water, the solution was titrated with Na_2_SO_3_ until the yellow color faded. Starch indicator was added, and the titration was continued until the blue color disappeared. A blank determination was conducted, and the PV (mEq/kg) was calculated using the following equation:

PV = ((*Vs* – *Vb*) × *N* × 1000)/*W* (1)

where: *Vs* is the volume of Na_2_SO_3_ used in the cooking oil sample until the yellow color faded (mL), *Vb* is the volume of Na_2_SO_3_ used in the blank sample until the yellow color faded (mL), *N* is the normality of Na_2_SO_3_ (mEq/mL used for titration), *W* is the weight of the cooking oil sample (g).

To measure free fatty acid (FFA), the percentage of free fatty acid in each sample was determined by the titration method (AOCS Ca 5a-40) [10]. Ten grams of sample were weighed into a flask and then neutralized with 50 mL of 95% ethanol and 1% phenolphthalein indicator. The mixture solution was heated to a maximum of 22 °C in a steam bath for 3 min, and then 2–3 drops of 1% phenolphthalein indicator were added. The final solution was titrated against sodium hydroxide solution (0.01 N) until a permanent pink color persisted for at least 30 s. The FFA (%) was calculated using the formula as follows:

FFA = (*V* × *N*(NaOH) × 25.6)/*W* (2)

where: *V* is the volume of NaOH used in the blank sample until the pink color persisted (mL), *N(NaOH)* is the normality of NaOH, *W* is the weight of the cooking oil sample (g).

### 2.2. Fortification of Cooking Oil and Vitamin A Analysis

Three cooking oils with different initial PV were fortified with vitamin A: (i) cooking oil of initial PV of 0 mEq O_2_/kg referred to as PO1 in this paper; (ii) PV of 4 mEq O_2_/kg (referred to as PO2); and (iii) PV of 9 mEq O_2_/kg (referred to as PO3).

The vitamin A premix, containing retinyl palmitate (1,700,000 IU/g), was procured through the GAIN (the Global Alliance for Improved Nutrition) premix facility (Geneva, Switzerland) [11] and added to the cooking oil in a laboratory (cooking oil was bought at the market and is representative of unbranded oil). The mixing process was conducted in a dark room at room temperature, and the vitamin A-fortified cooking oil was then analyzed for its homogeneity. The oil was then blown by nitrogen gas, sealed in closed amber bottles and stored in a dark room (inside an incubator) at elevated stress conditions (storage temperatures of 60, 75 and 90 °C) to determine their shelf life for a certain period of time.

Vitamin A levels were analyzed periodically using the method AOAC 2001.13 of the Official Analytical Chemists Association [12]. To determine vitamin A concentration, products are saponified in a basic ethanol-water solution, neutralized and diluted, converting fats to fatty acids and retinol esters to retinol. Retinol is quantified with a high-pressure liquid chromatography system with UV detection at 325 nm. Vitamin A concentration (C_vitaminA_, µg/g as retinol) is calculated by comparing peak heights or peak areas of vitamins in test samples with controlled standards using the following equation:

C_vitaminA_ = (RFA × *Pk* × 100)/*W* (3)

where: RFA is the response factor for vitamin A, *Pk* is the total test sample peak height or area of all *trans*- and 13-*cis* retinol, 100 is the dilution volume of the test portion (mL), *W* is the weight of the test portion (g).

### 2.3. Shelf Life Calculation of the Different Cooking Oil

The Arrhenius model was used to simulate the degradation of the oil stored under average Indonesian temperatures (30 °C ± 5 °C) [13] and to estimate the time until: (i) the PV reaches the maximum limit recommended by the Codex Alimentarius in the appendix for refined cooking oil for human consumption (10 mEq O_2_/kg) [14]; and (ii) the vitamin A reaches the minimum allowed by the national fortification standards (45 IU/g) [15]. The chemical reaction rate, which triggers food degradation, commonly follows a zero- and first-order reaction [16,17] for PV and vitamin A degradation, respectively, using the following equations:

*t*_s_ = (*Q*_0_ – *Q*_t_)/k (order 0 reaction) (4)

*t*_s_ = ln (*Q*_0_/*Q*_t_)/k (order 1 reaction) (5)

where: *t*_s_ is the shelf life (h), *Q*_0_ is the initial value of a quality, *Q_t_* is the acceptable final value of a quality, k is the constant of the reaction rate.

To obtain the constant of the reaction rate (k) of the palm cooking oil with different PV during storage at various temperatures, the Arrhenius equation is used as follows:

ln k = ln k_0_ − (E_a_/R) × (1/*T*) (6)

where: k is the constant of reaction rate of the oil affected by temperature, k_0_ is the pre-exponential factor, Ea is the activation energy, R is the universal gas constant (1.986 cal/mol K), *T* is the absolute temperature (in kelvins).

## 3. Results

The chemical characteristics of the three unbranded cooking oils are presented in Table 1 showing the different fortification levels and oxidative level.

**Table 1:** Chemical characteristics of unbranded cooking oil (average ± standard of deviation).

Cooking Oil Sample	Peroxide Value (mEq O_2_/kg)	Free Fatty Acid (%)	Retinyl Palmitate (IU/g)
PO1	0.000 ± 0.000	0.090 ± 0.000	55.85 ± 5.42
PO2	3.995 ± 0.002	0.238 ± 0.003	51.36 ± 1.28
PO3	8.987 ± 0.005	0.254 ± 0.005	67.49 ± 8.35

**Figure 1:**
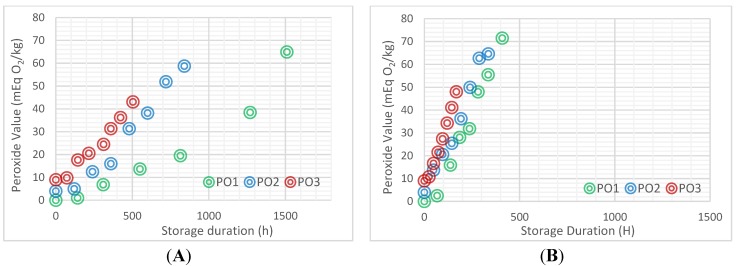

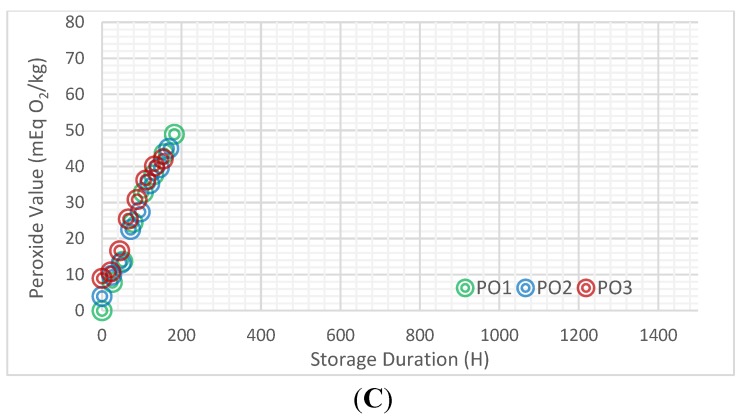
Peroxide formation in the three cooking oil samples with different initial peroxide value (PV) stored at 60 °C (**A**), 75 °C (**B**) and 90 °C (**C**).

The oxidative status of the cooking oils over time at 60 °C ± 5 °C (Figure 1A) showed a gradual increase in PV over time. The results of accelerated storage showed that the PV reached 10 mEq O_2_/kg within the 13th and 23rd day of storage for PO1, 5th and 10th day for PO2 and after only three days for PO3. Figure 1B,C shows that the level of peroxide is reached within 48 h for PO2 and PO3 at higher temperatures.

To reach the unacceptable PV of 10 mEq O_2_/kg of oil, the estimated shelf life of the fortified cooking oil is between two days to three months according to the initial PV (Table 2).

**Figure 2:**
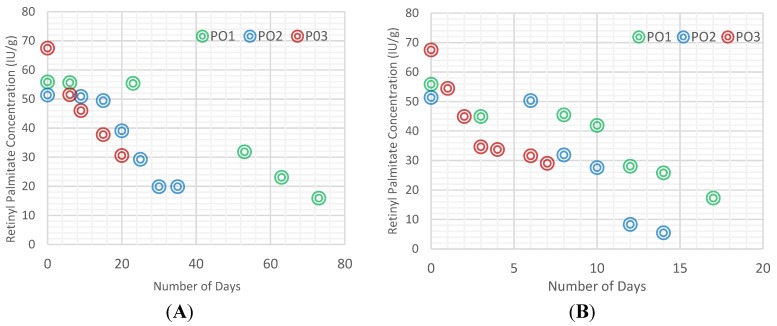

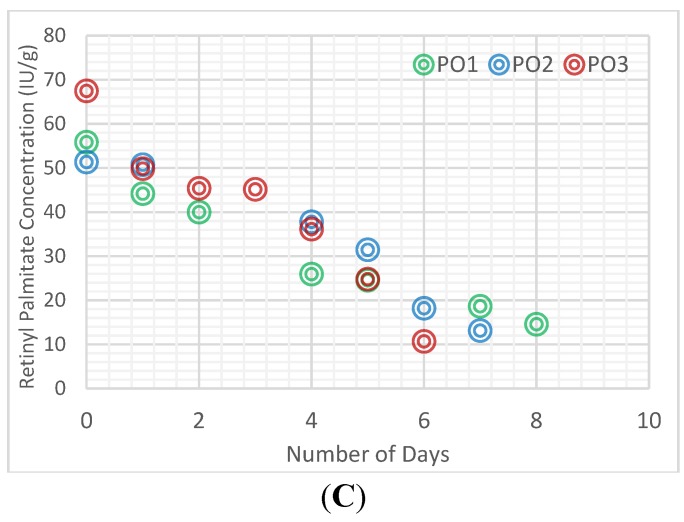
Retinyl palmitate degradation in the three cooking oil samples with different initial PV stored at 60 °C (**A**), 75 °C (**B**) and 90 °C (**C**).

During the accelerated test at 60 °C, the stability of vitamin A added to cooking oil was tested. Figure 2A shows that PO1 and PO2 lost 43% of its vitamin A content within 53 and 25 days, respectively, whereas PO3 lost the same amount of vitamin A in less than two weeks. In addition, the slopes of the concentration of vitamin A over time indicate an almost two- to three-fold higher deterioration of vitamin A in PO2 and PO3 compared to PO1, with slopes, respectively, of, −1.09, −1.79 and −0.57. At higher temperatures (75 °C, Figure 2B), half of the vitamin A was lost within 12 days for PO1, 10 days for PO2 and 3 days for PO3. The slopes of the concentration of vitamin A over time with a temperature of 75 °C and 90 °C also point out the deterioration of vitamin A between PO1 and PO3, with slopes, respectively, of −4.97 *vs.* −2.13 at 75 °C and −8.20 *vs.* −4.88 at 90 °C.

**Table 2:** Estimated shelf life of fortified cooking oils of different initial PVs according to the peroxide maximum (10 mEq O_2_/kg) during storage at 30 °C ± 5 °C in a dark room.

Q_0_ (PVi mEq O_2_/kg oil)	Q*_t_* (Final PV mEq O_2_/kg oil)	k (h^−1^)	*t*_s_ (Shelf Life)
0.00	10	4.59 × 10^−3^	2176.51 h (90.69 days or 3.02 months)
3.99	10	1.22 × 10^−2^	492.34 h (20.51 days or 0.68 months)
8.99	10	1.51 × 10^−2^	67.23 h (2.8 days)

PV was used as an indicator of shelf life. Table 3 presents the estimated final level of vitamin A in the three cooking oil samples at the time that the peroxide level reached 10 mEq/g (based on Table 2). Table 3 shows that less than 10% of the retinyl palmitate was lost by the time the cooking oils reached their shelf life.

**Table 3:** Estimated concentration of retinyl palmitate in fortified cooking oil of different initial PVs once they have reached their maximum PV (10 mEq O_2_/kg) during storage at 30 °C ± 5 °C in a dark room.

Cooking Oil Sample	k (h^−1^)	*t*_s_ (h)	Q_0_ (Vitamin A IU/g)	Q_t_ (Vitamin A IU/g)
PO1	3.02 × 10^−5^	2176.51 (3 months)	45	42.1
PO2	1.25 × 10^−4^	492.34 (0.6 months)	45	42.3
PO3	1.90 × 10^−4^	67.22 (2.8 days)	45	44.4

Note: *t*_s_ represents the time that the cooking oil can be kept for storage until the cooking oil reaches 10 mEq O_2_/kg, as shown in Table 2.

## 4. Discussion

Our study found that cooking oils in Indonesia have a shelf life between three days and three months before they reach the PV standard of less than 10 mEq O_2_/kg for cooking oil, as recommended by the appendix of the Codex Alimentarius [14]. The rancidity of cooking oil at the production level must also be taken into account, as peroxides can become carcinogens [18]. When it enters the human body in food, reactive oxygen species (ROS) and such other free radicals produced during lipid peroxidation can increase the risk of cancer and other chronic diseases. Soekirman *et al.* [1] showed that the average time between production and consumption of unbranded cooking oil is three weeks, and therefore, according to our results, the maximum recommended peroxide level would be reached. Only cooking oil with the lowest initial PV (PO1) would be fit for consumption.

Fortifying unbranded oil with a high PV (around 9 mEq O_2_/kg) could have as much of a negative health impact (by increasing ROS and other end products of lipid peroxidation, a cause of many chronic health problems, such as cardiovascular and inflammatory disease, cataracts and cancer [19]) as a positive one (preventing and reducing vitamin A deficiency). It is therefore essential that government/regulatory bodies take measures to forbid the production and sale of highly-oxidized cooking oils. Such measures will result in a longer shelf life for cooking oil and higher stability of cooking oil, as seen previously in Egypt [4]. However, highly-oxidized cooking oils are not widely observed in Indonesia. In a 2012 study implemented by the SEAFAST Center [20], most of the unbranded cooking oil collected had a mean PV of 1.14 mEq O_2_/kg in Java and 3.33 mEq O_2_/kg in Sumatera. Such cooking oils, according to our analysis, could be kept at least one month, which is within the observed timeframe from production to consumption in Indonesia [1].

In our study, vitamin A loss at the time the oil reached its maximum acceptable value for peroxide (10 mEq O_2_/kg) was still at an acceptable level of less than 10% of the national standard at the production site (45 IU/g). However, degradation of vitamin A in sample PO3 was faster than in the two other oils (PO1 and PO2) shown by its highest reaction rate constant (1.90 × 10^−4^/h). These results are consistent with research conducted by Laillou *et al.* [4], who added vitamin A into edible cooking oils with two different initial PVs (5.8 and 0.4 mEq O_2_/kg oil, stored at 30 °C ± 5 °C). Laillou also found that vitamin A losses started when the PV reached a level of approximately 2 mEq O_2_/kg and were closely associated with the increase of peroxides [4]. Our study is in line with this study and a recent one published by Pignitter *et al.* [21]. This more recent study reported that either mildly- or highly-oxidized soybean oil samples filled in the transparent bottle and exposed to the cold fluorescence light at 22 or 32 °C for 56 days underwent the retinyl palmitate loss up to 84.8% ± 5.76% by an increase of the peroxide value from 1.20 ± 0.004 to 24.3 ± 0.02 mEq O_2_/kg. During our study, we used several temperatures to accelerate the process. The temperatures were selected to stimulate relatively fast degradation, to determine the shelf life using ASLT. Therefore, temperature is the only environmental factor that was investigated. The light exposure and oxygen availability were avoided by storing the samples in amber bottles, flushed with nitrogen, and sealing and keeping them in the dark incubator. We can imagine that the addition of other negative environmental factors, such as light and long storage, could furthermore accelerate the increase of the peroxide value and the degradation of vitamin A, as was shown in other studies.

In summary, the findings of this study recommend that PV of bulk palm oil must be kept as low as possible to successfully accomplish vitamin A fortification in Indonesia and to also improve the quality of cooking oil for lengthier storage shelf lives at room temperature (if kept in dark, as well), without jeopardizing health outcomes.

## 5. Conclusions

Indonesia has the potential to reach its entire population with cooking oil fortified with vitamin A, even its most vulnerable groups in need of this important vitamin. However, it is essential to fortify only cooking oils that meet certain basic quality criteria. Our study shows that unbranded cooking oil with a high initial PV has a very short shelf life and will quickly become unfit for human consumption. To protect human health, the Indonesian government should prohibit highly-oxidized cooking oil from being sold on the market. In addition, the government should promote and endorse the fortification of only cooking oil with low peroxide levels to ensure that fortification is not wrongly associated with health issues caused by the oxidation level of cooking oil.
